# SADQN-Based Residual Energy-Aware Beamforming for LoRa-Enabled RF Energy Harvesting for Disaster-Tolerant Underground Mining Networks

**DOI:** 10.3390/s26020730

**Published:** 2026-01-21

**Authors:** Hilary Kelechi Anabi, Samuel Frimpong, Sanjay Madria

**Affiliations:** 1 Department of Mining and Explosives Engineering, Missouri University of Science and Technology, Rolla, MO 65409, USA; frimpong@mst.edu; 2 Department of Computer Science, Missouri University of Science and Technology, Rolla, MO 65409, USA; madrias@mst.edu

**Keywords:** adaptive beamforming, energy beamforming (EB), Safe Deep Q-Network (Safe-DQN), energy harvesting (EH), Long Range (LoRa), wireless power transfer (WPT), reinforcement learning (RL)

## Abstract

The end-to-end efficiency of radio-frequency (RF)-powered wireless communication networks (WPCNs) in post-disaster underground mine environments can be enhanced through adaptive beamforming. The primary challenges in such scenarios include (i) identifying the most energy-constrained nodes, i.e., nodes with the lowest residual energy to prevent the loss of tracking and localization functionality; (ii) avoiding reliance on the computationally intensive channel state information (CSI) acquisition process; and (iii) ensuring long-range RF wireless power transfer (LoRa-RFWPT). To address these issues, this paper introduces an adaptive and safety-aware deep reinforcement learning (DRL) framework for energy beamforming in LoRa-enabled underground disaster networks. Specifically, we develop a Safe Adaptive Deep Q-Network (SADQN) that incorporates residual energy awareness to enhance energy harvesting under mobility, while also formulating a SADQN approach with dual-variable updates to mitigate constraint violations associated with fairness, minimum energy thresholds, duty cycle, and uplink utilization. A mathematical model is proposed to capture the dynamics of post-disaster underground mine environments, and the problem is formulated as a constrained Markov decision process (CMDP). To address the inherent NP hardness of this constrained reinforcement learning (CRL) formulation, we employ a Lagrangian relaxation technique to reduce complexity and derive near-optimal solutions. Comprehensive simulation results demonstrate that SADQN significantly outperforms all baseline algorithms: increasing cumulative harvested energy by approximately 11% versus DQN, 15% versus Safe-DQN, and 40% versus PSO, and achieving substantial gains over random beamforming and non-beamforming approaches. The proposed SADQN framework maintains fairness indices above 0.90, converges 27% faster than Safe-DQN and 43% faster than standard DQN in terms of episodes, and demonstrates superior stability, with 33% lower performance variance than Safe-DQN and 66% lower than DQN after convergence, making it particularly suitable for safety-critical underground mining disaster scenarios where reliable energy delivery and operational stability are paramount.

## 1. Introduction

Traditional battery-powered tracking and localization devices face fundamental energy limitations in post-disaster underground mine scenarios [1]. Wireless power transfer (WPT) offers a valuable path to sustain these nodes, but underground propagation is highly lossy and time-varying due to attenuation, irregular tunnel geometry, obstructions, and multipath, which can sharply reduce the end-to-end radio-frequency WPT (RF-WPT) efficiency [2]. The feasibility of deploying radio-frequency energy harvesting (RF-EH) in underground mining environments has been demonstrated empirically with appropriate technical adjustments [3]. However, the end-to-end RF-WPT efficiency in underground mines remains substantially low, necessitating adaptive techniques for optimizing WPT in such complex and rapidly changing environments [4]. Efficient techniques such as waveform optimization, energy beamforming (EB), and distributed antenna systems are therefore essential to achieve the optimal performance of underground RF-WPT [5,6].

Energy beamforming (EB) has emerged as an innovative technique to enhance the efficiency and range of WPT systems by steering concentrated beams of energy toward one or more energy harvesting (EH) devices simultaneously. Through coordinated antenna control, EB focuses RF energy on target devices, limiting dispersion, lowering path loss, and improving spatial filtering. This targeted power delivery greatly improves transfer efficiency, making EB especially valuable in environments with dynamic channels, severe multipath fading, and limited coverage conditions that are endemic to underground mining. As a result, EB provides a promising pathway to achieve reliable and energy-efficient wireless power transfer in complex underground mine settings where conventional omnidirectional transmission techniques often fall short [7,8].

Conventional EB systems are designed under the assumption of perfect channel state information (CSI) to achieve the optimal beamforming performance. However, obtaining accurate CSI is often impractical, particularly in dynamic and infrastructure-limited environments where frequent channel variations, multipath effects, and limited feedback mechanisms make real-time estimation highly challenging. These challenges are exacerbated in post-disaster underground mining scenarios where communication infrastructure may be partially destroyed, feedback channels unreliable, and rapid environmental changes render CSI outdated almost immediately. To address these limitations, approaches based on convex optimization and deep reinforcement learning (DRL) have shown significant promise, enabling EB systems to adaptively optimize beam directions and power allocation while maintaining high energy transfer efficiency even in complex and rapidly changing wireless environments [9,10,11]. More recently, DRL has emerged as a powerful alternative, enabling autonomous decision-making and learning optimal beamforming strategies directly from environmental interactions, making it particularly effective for EB applications in scenarios with imperfect or time-varying CSI [12].

By interacting with the environment, DRL agents learn optimal policies from environmental dynamics to perform complex tasks without explicit programming and modeling. DRL methods address several limitations of conventional techniques by providing real-time, online learning solutions capable of operating in highly dynamic and uncertain environments [13,14]. Compared to conventional beamforming techniques, DRL-based frameworks are better suited for time-sensitive and resource-constrained applications due to their scalability, policy adaptability, and ability to learn directly from environmental interactions without requiring complete system models or perfect CSI. Despite significant progress in both adaptive beamforming and DRL-based wireless optimization, existing approaches suffer from three critical limitations when applied to underground disaster scenarios [15,16,17].

First, they lack explicit safety mechanisms. In post-disaster mining networks, violating operational constraints can have catastrophic consequences: allowing nodes to drop below minimum energy thresholds means losing tracking capability for trapped miners; and unfair energy distribution may leave some nodes powerless while others are over-provisioned. Second, existing methods do not prioritize nodes based on residual energy awareness. Most beamforming algorithms treat all energy-harvesting nodes equally or optimize for aggregate metrics like sum energy or minimum energy across all nodes. Third, they do not address the unique requirements of long-range, low-power communication. Post-disaster underground environments demand communication technologies that can maintain connectivity over extended distances through debris, collapsed structures, and hostile RF conditions. Long-range (LoRa) technology, with its sub-GHz operation, spread-spectrum modulation, and ability to achieve ranges exceeding 10 km in favorable conditions [18,19], is uniquely positioned for this application given its extremely low power consumption (−140 dBm receiver sensitivity).

The problem of jointly optimizing beamforming direction, energy allocation, and constraint satisfaction in underground disaster networks is fundamentally an NP-hard constrained optimization problem. This computational complexity is particularly problematic in disaster scenarios where decisions must be made in real-time on resource-constrained embedded hardware without external connectivity to cloud computing resources. To address these critical gaps, this work first proposes the Safe Deep Q-Network (Safe-DQN)-based energy beamforming framework tailored specifically for underground mining disaster scenarios. Unlike standard DQN-based beamforming strategies that optimize solely for energy delivery, the proposed method explicitly incorporates operational constraints such as minimum voltage thresholds, fairness among EH nodes, duty-cycle limits, and uplink utilization bounds directly into the learning objective using Lagrangian penalized reward functions, enabling the framework to maximize harvested energy while ensuring system safety, operational reliability, and stability in harsh underground wireless environments. Safe-DQN is not an adaptive algorithm, and hence, it is limited.

We then proposed Safe Adaptive DQN (SADQN), which extends the baseline Safe-DQN by incorporating residual energy-aware scoring that prioritizes the weakest nodes those at risk of failure, and adaptive exploration mechanisms that dynamically adjust exploration rates based on system conditions: increasing exploration when energy distribution is highly unbalanced and prioritizing exploitation when the system is stable and fair. The major contributions of this work include the following:

- Safe-RL formulation for energy beamforming: We model beamforming as a constrained Markov decision process (CMDP) with sequential charging of the weakest node and penalties for unsafe beam-switching, voltage drops, fairness violations, and excessive duty-cycle usage, employing Lagrangian relaxation with dual-variable updates to address the NP hardness of this problem.
- Residual energy-aware adaptive mechanisms: We introduce energy-aware scoring that prioritizes nodes with the lowest residual energy, combined with fairness-aware top-K action selection and adaptive exploration that responds to system stability.
- LoRa voltage feedback for beam optimization: To the best of our knowledge, this is the first work to use real-time LoRa voltage feedback as the observable state for reinforcement learning in RF-WPT systems, with a dual-band architecture (860 MHz for WPT, 915 MHz for LoRa uplink) enabling long-range, low-power feedback without requiring extensive CSI acquisition.

The rest of the paper is organized as follows: Section 2 presents the related studies. Next, in Section 3, we present our system model and the optimization problem. In Section 4, we discuss our proposed solution, while Section 5 provides numerical results. Finally, Section 6 concludes the work.

## 2. Related Studies

EB has recently gained renewed attention as a critical enabler of high-efficiency wireless power transfer for energy-constrained nodes, particularly in challenging environments such as underground mines and disaster-prone areas. Since 2022, there has been a notable shift toward integrating machine learning, hardware-efficient beamforming, and low-CSI paradigms to improve system robustness and adaptability. This section reviews recent advances across four key areas: energy beamforming techniques, reinforcement learning for wireless systems, safe and constrained optimization, and LoRa-based underground communications. Recent work has focused on improving beamforming efficiency through novel hardware architectures and CSI-reduction techniques. For instance, Azarbahram et al. (2024) designed an energy beamforming solution using Dynamic Metasurface Antenna (DMA) transmitters, which minimize transmit power while maintaining energy delivery constraints [20]. Unlike conventional fully digital or hybrid arrays, their approach enables frequency-domain beam shaping at a reduced RF chain cost, achieving a 40% improvement in energy efficiency for the same coverage. However, their work assumes static propagation environments and perfect knowledge of user locations, limiting applicability to dynamic disaster scenarios where infrastructure may be compromised. Similarly, Zhang et al. (2024) proposed a two-stage beamforming protocol leveraging radar-like sensing to extract path direction and gain without requiring explicit CSI feedback [16]. Their method closely matches the performance of full CSI-based beamforming, making it a promising solution for feedback-constrained and underground deployment scenarios. However, it does not address energy-aware node prioritization or operational safety constraints critical in life-threatening situations.

Yang, Zhang, and Wang (2022) introduced a joint optimization framework for simultaneous radar sensing and WPT [17], demonstrating the feasibility of dual-function antenna systems for 6G networks. While innovative, this work optimizes for sensing-WPT trade-offs rather than fairness, minimum energy guarantees, or node survival essential requirements in disaster response operations. RL has been increasingly integrated into beamforming research to address uncertainty in dynamic wireless environments. Joint optimization of trajectory, beamforming, and power allocation in UAV-enabled WPT networks has been explored using DRL combined with water-filling algorithms [21], enabling UAVs to dynamically adjust flight paths to maximize the minimum harvested energy across users. Narengerile et al. (2022) applied DRL for mmWave beam training, achieving improved spectral and energy efficiency under real-time dynamics [22]. Zhou et al. [23] proposed a DQN-based algorithm to improve spectral efficiency in mmWave systems under train mobility, while Ge et al. [24] utilized a deep deterministic policy gradient (DDPG) approach for optimizing the sum rate in multi-user MISO systems. Dantas et al. [25], introduced actor–critic models to address beam selection in fast-varying wireless environments.

Although these contributions demonstrate significant progress, they share critical limitations when applied to underground disaster scenarios. The majority focus on maximizing data throughput (spectral efficiency, sum rate) rather than optimizing wireless energy transfer and node survivability. They lack explicit safety mechanisms, failing to incorporate hard constraints on minimum energy thresholds, fairness guarantees, or duty-cycle compliance essential to prevent catastrophic node failures during rescue operations. Most assume availability of CSI or stable feedback channels, which may be unreliable or unavailable when communication infrastructure is compromised. Finally, none addresses the unique challenge of long-range, low-power communication required in post-disaster underground environments.

Safe reinforcement learning extends conventional RL by incorporating operational constraints to guarantee an acceptable performance during both learning and deployment. While safe RL has been applied to robotics and autonomous systems, its application to wireless energy transfer remains limited. Zhang et al. [26] applied Q-learning to distribute RF energy fairly among IoT devices, and Mao et al. [27] developed an RL-based dynamic charging scheduler to optimize wireless power delivery under varying network conditions. However, these approaches optimize for fairness or charging efficiency without explicitly enforcing hard safety constraints such as minimum voltage thresholds, beam-switching stability, or regulatory duty-cycle limits. Zhang et al. [28] explored multi-agent reinforcement learning (MARL) for mobile charger path optimization in wireless sensor networks. While effective for temporal scheduling, these models assume ideal channels or centralized control, lacking spatial selectivity and adaptability to channel impairments such as obstruction, multipath fading, and dynamic interference characteristic of underground mines. Moreover, existing energy-aware RL methods do not implement residual energy-based prioritization, a triage principle essential for disaster scenarios where the most energy-starved nodes must be charged first to maintain tracking and localization of trapped miners.

In the domain of LoRa-based wireless energy harvesting, ref. [19] examined LoRa network performance under ambient energy harvesting and random transmission schemes, demonstrating feasibility but not addressing directional energy delivery or adaptive beamforming. Emmanuel [18] characterized LoRa signal behavior in underground tunnels, providing valuable propagation insights but without integrating wireless power transfer. Kumar et al. [29], presented robust gas-monitoring devices leveraging LoRa for Through-The-Earth (TTE) communication, validated under non-line-of-sight underground conditions, and designed a disaster-tolerant LoRa communication framework tailored for underground mine environments. While these studies emphasize connectivity, reliability, and environmental robustness, they do not address power sustainability, directional energy delivery, or learning-based adaptive control. None explore the dual-band architecture required to simultaneously support RF energy transfer and LoRa-based feedback, nor do they address the 125+ dB power asymmetry between energy-harvesting requirements (−15 dBm minimum) and LoRa communication sensitivity (−140 dBm). Table 1 summarizes the key differences between prior work and our proposed approach. In contrast, our work introduces a Safe Adaptive Deep Q-Network (SADQN) energy-beaming framework for RF energy delivery to LoRa-enabled energy-harvesting nodes in underground disaster environments.

## 3. System Model

Conventional beamformers, while simple and effective, perform poorly in environments with multiple signal directions, high noise levels, and dynamic channel conditions typical of post-disaster underground mines. To address these challenges, adaptive beamforming techniques dynamically adjust beam weights based on real-time environmental conditions, enabling robust operation in complex and rapidly changing scenarios. In this section, we present our adaptive energy beamforming system model for underground mine disaster environments. Some of the symbols and meanings are shown in Table 2.

### 3.1. System Architecture and Network Model

**Figure 1:**
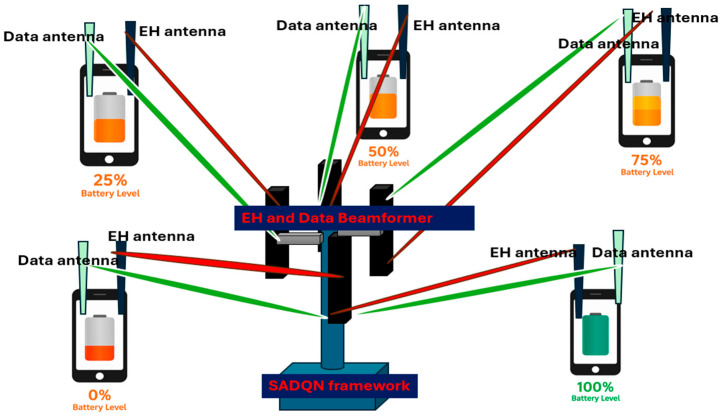
The EB-based WPT system architecture.

We consider a multi-antenna RF wireless power transfer (RF-WPT) system operating in an underground mine environment, where a transmitter is equipped with a uniform linear array (ULA) and $N_{t}$ antennas deliver RF energy to uniform linear array *N* LoRa EH double-antenna nodes, as depicted in Figure 1. Each LoRa EH node is equipped with a dual-antenna configuration: one antenna dedicated to RF energy harvesting and the other to LoRa-based wireless communication. This design allows nodes to sustain energy harvesting and data transmission concurrently, ensuring continuous operation in energy-constrained underground environments. To avoid cross-interference between the two functions, separate frequency bands are employed. Specifically, 860 MHz is selected for RF energy harvesting $f_{WPT}$, following our prior experimental validation in underground mine environments [3], while the U.S. LoRa frequency $f_{LoRa}$ of 915 MHz is adopted for uplink communication. This dual-band architecture enables real-time voltage feedback from energy-starved nodes without interfering with the downlink power transfer process. It is important to note that RF energy harvesting imposes stringent requirements on received signal power. For practical EH operation, the received RF power must be at least −15 dBm [3], whereas reliable LoRa information decoding can be sustained at power levels as low as −140 dBm. This 125 dB disparity highlights the fundamental challenge of RF-EH in underground mine environments, which are inherently lossy due to multipath fading, shadowing, and complex dielectric properties of geological materials.

### 3.2. Underground Mine Propagation Model

The underground mine wireless propagation channel is characteristically different from other legacy wireless environments. Node *i* received power in (dBm) from a WPT transmitter placed at a distance $d_{i}$ that can be estimated using Equation (1):

(1) $$P_{i,j}^{r}\;=P_{T}-PL\left(d_{ij}\right)+X_{\sigma ,\;}\;$$

$P_{T}$ denotes the total radiated transmit power including the contribution of the external power amplifier (PA), PL(.) is the environment calibrated path loss term, and $X_{\sigma \;}$ represents log-normal shadowing in dB, typically, 3–10 dB in underground settings. Underground mines employ a specific path loss model accounting for tunnel geometry [30],

(2) $$PL\left(d\right)=PL\left(d_{0}\right)+10\;n_{Corridor}\log_{10}\left(d/d_{0}\right)+N_{Wall}\cdot L_{Wall}+{{\;N}_{Junction\cdot }L}_{Junction}+L_{Moist},\;$$

- $PL\left(d_{0}\right)=40\;\mathrm{dB}$: the reference path loss at $d_{0}=1m$.
- $n_{Corridor}=2.2$: the corridor propagation exponent deduced from free space due to waveguide effects.
- $L_{Wall}=25$ dB: additional loss per rock wall or rough surface penetration.
- $L_{Junction}=8$ dB: scattering loss per tunnel intersection or crosscut.
- $L_{Moist}$ = 2–5 dB: absorption due to water vapor in mine air.
- $N_{Wall}$, $N_{Junction}$: number of walls and junctions traversed along the propagation path.

This model captures the unique waveguide-like propagation in underground tunnels, where reflections from walls can either constructively or destructively interfere with the direct signal. Equation (2) is a combination of a basic pathloss formula and underground-specific pathloss component, as stated in [30].

### 3.3. Channel Fading Model

The wireless channel between the transmitter and each node experiences both deterministic and stochastic components. We model the channel as Rician fading, which captures both the line-of-sight (LoS) component and scattered non-line-of-sight (NLos) components. The baseband channel coefficient for node *i* is expressed as in Equation (3):

(3) $$h_{i}\left(t\right)=\sqrt{\frac{K}{K+1}}\cdot h_{LoS}+\sqrt{\frac{1}{K+1}}\cdot h_{NLoS,\;}$$

K is the Rician K-factor, defined as the power ratio between the LoS and NLos components. In underground mine environments, K typically ranges from 3 to 10 dB, depending on tunnel geometry and the presence of obstructions. The term $h_{Los}$ denotes the deterministic LoS component, while $h_{NLos}\left(t\right)\sim CN\left(0,I\right)$ represents Rayleigh distributed scattering with independent and identically distributed (i.i.d.) complex Gaussian entries. In the special case where the LoS path is completely blocked (e.g., by collapsed debris), the model reduces to pure Rayleigh fading with K=0 as given by Equation (4):

(4) $${\;h_{i}\left(t\right)=h}_{NLos}\left(t\right)\sim CN\left(0,I\right),\;$$

Underground mine openings are predominantly tunnel-like structures with maze-like interconnections. The confined geometry makes the Rician model particularly appropriate, as it captures both the strong guided LoS component and the scattered multipath caused by reflections and diffractions from tunnel walls.

### 3.4. Received Power and Beamforming Gain

Combining the propagation and fading models, the instantaneous received power at node *i* at time *t* is expressed as Equation (5):

(5) $${\;P}_{i,r}^{t}=P_{T}\cdot G\left({\;\theta }_{t}\right)\cdot {\left|h_{i}\right|}^{2}\cdot {\left(\frac{\lambda }{4\pi d_{i}}\right)}^{2}\cdot \frac{1}{L\left(d_{i}\right)},\;$$

- $P_{T}$ is the total transmit power (including PA gain).
- $G\left(\theta_{t}\right)$ is the beamforming gain in the direction of node *i.*
- $h_{i}$ is the instantaneous channel gain (from Equation (3) or (4)).
- ${\left(\lambda /4\pi d_{i}\right)}^{2}$ is the free-space path loss term.
- $1/L\left(d_{i}\right)=10^{PL\left(d_{i}\right)/10}$ converts the dB path loss from Equation (2) to a linear scale.

This formulation integrates the array gain, channel fading, distance-dependent path loss, and underground-specific attenuation into a unified received power expression.

### 3.5. Steering Vector and Beam Gain Model

To realize directional RF energy transfer, the transmitter employs an array steering vector a(θ) to form a beam pattern corresponding to the desired azimuth angle $\theta_{t}$ in 2D. For a uniform linear array (ULA) with $N_{t}$ elements and inter-element spacing *d*, the array manifold vector is expressed as Equation (6):

(6) $$a\left(\theta \right)={\left[1\;e^{jkd\;sin\left(\theta \right)}\ldots e^{\left(N_{t}-1\right)jkd\;sin\left(\theta \right)}\right]}^{T},\;$$

k=2π/λ is the wavenumber associated with the carrier wavelength λ, and θ is the azimuth angle (in radians) measured from broadside. The beamforming objective is to steer the transmit beam toward a designated direction ${\;\theta }_{0}.$ This is achieved using conjugate beamforming with unit-norm weight vectors:

(7) $$w\left(\theta_{0}\right)=\frac{a^{*}\left(\theta_{0}\right)}{\left\|a\left(\theta_{0}\right)\right\|}=\;\frac{1}{N_{t}}{\left[1\;e^{-jkd\;sin\left(\theta_{0}\right)}\ldots e^{-j\left(N_{t}-1\right)kd\;sin\left(\theta_{0}\right)}\right]}^{T},\;$$

When steering toward θ, a conjugate phase shift is applied across the array elements such that radiated waves combine coherently in that direction. The corresponding array response is given by Equation (8):

(8) $$H\left(\theta ;\theta_{0}\right)=w^{H}\left(\theta_{0}\right)a\left(\theta \right)\;=\;\frac{1}{\sqrt{N_{t}}}\sum_{n=0}^{N_{t}-1}e^{jnkd\;\left(sin\theta -sin\theta_{0}\right)},\;$$

The associated power gain is the squared magnitude of this response, given by Equation (9):

(9) $${G\left(\theta ;\theta_{0}\right)=\left|H\left(\theta ;\theta_{0}\right)\right|}^{2}\;=\frac{1}{N_{t}}{\left|\sum_{n=0}^{N_{t}-1}e^{jkd\;\left(sin\theta -sin\theta_{0}\right)}\;\right|}^{2},\;$$

It is evident from (8) that the maximum beamforming gain occurs under perfect alignment, i.e., when $\theta =\theta_{0}.$ In this case, the array achieves coherent combining, and the gain scales linearly with the number of antennas. The beamforming gain $G\left(\theta ;\theta_{0}\right)$ quantifies the power amplification achieved by the antenna array when steered toward direction $\theta_{0}$ and evaluating the response at angle θ. It represents the radiated power intensity in direction θ to the average radiated power intensity that would result from an isotropic radiator with the same total input power.

#### 3.5.1. Perfect Alignment $\left(\theta =\theta_{0}\right)$

Under perfect alignment, all array elements contribute constructively, yielding maximum gain in Equation (10):

(10) $$\left(\theta ;\theta_{0}\right)\;=\frac{1}{N_{t}}{\left|\sum_{n=0}^{N_{t}-1}\;1\;\right|}^{2}=\frac{1}{N_{t}}\cdot N_{t}^{2}=N_{t}\;,\;$$

This shows that the peak beamforming gain scales linearly with the number of antenna elements. For example, with $N_{t}=16$ elements, the maximum gain is $G_{max}=16$ or equivalently $10\;\log_{10}\left(N_{t}\right)\;=10\;\log_{10}\left(16\right)\;\approx 12\;dB$.

#### 3.5.2. Off-Boresight Angles $\left(\theta \neq \theta_{0}\right)$

When the observation angle θ deviates from the steering angle $\theta_{0}$, the gain decreases due to destructive interference among array elements. The gain pattern exhibits sidelobes and nulls characteristic of array antenna radiation patterns.

### 3.6. Gauss–Markov Drift Mobility Model

In underground mine environments, node mobility and scattering-induced perturbations introduce fluctuations in the angle of arrival (AoA), which directly affect beam alignment and energy transfer efficiency. These variations are characterized by the instantaneous angular drift $\phi_{i,t}$ at time t modeled here using the Gauss–Markov drift process in Equation (11):

(11) $$\phi_{i,t+1}=a_{0}\phi_{i,t}+\left(1-a_{0}\right)\xi_{i,t},\;\xi_{i,t}\sim N\left(0,\sigma_{0}^{2}\right),\;$$

$a_{0}\in \left[0,1\right)$ is the temporal correlation coefficient. A value $a_{0}=0$ corresponds to uncorrelated white noise, while values approaching unity indicate highly correlated angular drift. The innovation term $\xi_{i,t}$ represents Gaussian perturbations, with the standard deviation set to $\sigma_{0}=\sigma_{rad}=\sigma_{drift}=0.002$ radians, modeling small mobility-induced deviations. In a steady state, the drift variance is given as Equation (12):

(12) $$Var\left[\emptyset \right]=\frac{\left(1-a_{0}\right)}{\left(1+a_{0}\right)}\sigma_{0}^{2},\;$$

and the temporal covariance at lag k is given by Equation (13):

(13) $$Cov\;\left(\phi_{t},\phi_{t+k}\right),\;=Var\left[\phi \right]\cdot a_{0}^{k}\;$$

The beam angle is then updated as Equation (14):

(14) $${\;\theta }_{i,t+1}=clip\left(\theta_{i,t}+\phi_{i,t+1},\theta_{min},\theta_{max}\right),\;$$

$\theta_{min}=-{\;60}^{{}^{\circ}}$ and $\theta_{max}={+\;60}^{{}^{\circ}}$ enforce the angular span constraint $\pm 60^{{}^{\circ}}$, corresponding to a total beam search space of $\theta_{span}{=120}^{{}^{\circ}}$. To also capture spatial displacement, node motion is represented by a two-dimensional Gaussian random walk, given by Equation (15):

(15) $$r_{i,t+1}=clip\left(r_{i,t}+\delta_{i,t},r_{min},r_{max}\right),$$

where $\delta_{i,t}\sim N\left(0,\sigma_{0}^{2}Ι\right)$ is the innovation noise introducing random perturbations into correlated motion. The displacement speed is bounded by $\omega_{max}=0.5\;m/s$ and a waypoint tolerance of ${∄}_{wp}=0.3\;m$, which ensures that a node is considered to have reached a waypoint once within this proximity.

### 3.7. RF Energy-Harvesting Storage Dynamics

We next model the RF-EH capacity of the LoRa EH device. Assuming a linear harvesting process, the energy storage dynamics are expressed as

(16) $$E_{i,t+1}={clip}_{\left[E_{min},E_{max}\right]}\left(E_{i,t}+\eta_{RF-DC}\left(P_{i,t}\right)\tau -\left(P_{i,t}^{load}\right)\tau -\left(\frac{E_{i,t}}{\rho_{leak}}\right)\tau \right)$$

where the operator ${clip}_{\left[E_{min},E_{max}\right]}$ constrains the stored energy to the feasible interval $\left[E_{min},E_{max}\right]$. The terms in Equation (16) represent the following:

- $E_{i,t}$: residual stored energy in the capacitor or battery (J).
- $\eta_{RF-DC}\left(P_{i,t}\right)\tau$: harvested RF energy with conversion efficiency (J).
- $\left(P_{i,t}^{load}\right)\tau$: energy consumed by the sensor, microcontroller, and LoRa transceiver load (J).
- $\left(\frac{E_{i,t}}{\rho_{leak}}\right)\tau$: leakage losses due to capacitor self-discharge or battery leakage current.

Here, τ is the energy transmission duration per time slot. The voltage at node *i* is related to stored energy, which is given by Equation (17):

(17) $${\;\nu }_{i,t}=\sqrt{\frac{{2E}_{i,t}}{C}},\;$$

C is the capacitance of the energy storage element. This voltage feedback is transmitted via the LoRa uplink and serves as the primary observable for the SADQN beamforming agent.

### 3.8. Beyond Far-Field Beamforming Propagation Region

The primary motivation for adopting RF-WPT in underground mine post-disaster scenarios lies in its ability to support far-field energy delivery, which is essential for powering tracking and localization systems when conventional infrastructure is compromised. The far-field boundary for an antenna array of maximum physical aperture size *D* operating wavelength $\lambda_{ο}$ [20] is given by Equation (18):

(18) $$d_{far}=\frac{{2D}^{2}}{\lambda_{ο}}\;,\;$$

For ULA with $N_{t}$ elements with inter-element spacing $d=\lambda_{ο}/2$, the effective aperture size is $D=\left(N_{t}-1\right)d$. When substituting this into (15), the far-field distance becomes Equation (19):

(19) $$d_{far}=2\frac{2{\left[\left(N_{t}-1\right)\lambda_{ο}/2\right]}^{2}}{\lambda_{ο}}=\frac{{\left(N_{t}-1\right)}^{2}\lambda_{ο}}{2}\;,$$

As a practical example, consider a UHF-based RF-WPT transmitter operating at $f_{WPT}\;$= 860 MHz, corresponding to a wavelength of $\lambda_{ο}\approx \;0.349\;m$. For ULA with $N_{t}=4$ elements, (18) yields a far-field distance of approximately 1.57 m. Consequently, a LoRa receiver must be positioned at least 1.57 m from the transmitting array for the far-field propagation assumption to remain valid. The overarching objective of this work is not merely to satisfy the minimum far-field condition but to enable efficient energy beamforming beyond the conventional far-field region, ensuring robust and reliable RF energy delivery throughout the complex underground mine environment. This far-field foundation underpins the design of our SADQN beamforming algorithm, which dynamically exploits angular steering to maximize harvested energy while satisfying safety constraints in underground disaster networks.

## 4. Safe Reinforcement Learning Problem Formulation

Safe RL is a branch of the general RL paradigm that, in addition to maximizing expected returns, explicitly incorporates safety constraints to guarantee an acceptable performance during both the learning and deployment phases. Unlike conventional RL frameworks that optimize solely for cumulative reward, Safe RL enforces operational limits to prevent unsafe or infeasible actions, making it particularly suited for safety-critical environments such as underground mine communications and RF energy transfer.

In this section, we present the details of our environment modeling, the baseline Deep Q-Network (DQN) formulation, and the associated constrained problem setup. Building upon this foundation, we develop the proposed Safe Adaptive DQN framework (SADQN), which extends the Safe-DQN architecture by introducing adaptive exploration and energy-aware constraint handling tailored to underground wireless power transfer scenarios.

### 4.1. Environment Modeling

The Markov decision process (MDP) provides a well-established mathematical framework for RL and DRL optimization problems [31]. A task consists of a set of related states, actions, and rewards [32]. In this work, we extend the MDP to a constrained Markov decision process (CMDP) to capture the safety-critical requirements of WPT in underground mine environments. The CMDP is defined by the tuple in Equation (20):

(20) M≝(S,A,R,P,γ,C,B), 

S denotes the state space, A is the action space, R the reward function, P the state transition probabilities, and γ the discount factor. In addition, C represents the cost constraint functions, and B the corresponding budget thresholds. Without safety constraints, the CMDP reduces to the standard MDP formulation (S,A,R,P,γ). The objective is to learn an optimal policy $\pi^{*}$ that maximizes the expected discounted return while satisfying the safety constraints given Equation (21):

(21) $$\pi^{*}=arg\;\underset{\pi }{\max\;}E_{\pi }\left[\sum_{t=0}^{\infty }\gamma^{t}r^{t}\right],\;$$

$r^{t}$ is the reward at time step *t*.

#### 4.1.1. State Space S

The state space S encapsulates all information required to characterize the environment at time *t*. For the LoRa-EH beamforming problem, the state vector is defined as Equation (22):

(22) st=[v¯tVmax(θt−1+π2)πJ¯t]∈R3, 

${\bar{v}}_{t}$ is the exponentially smoothed harvested voltage, $\theta_{t-1}$ is the previous beam angle, and ${\bar{J}}_{t}$ the smoothed Jain’s fairness index. The fairness metric is defined as Equation (23):

(23) $$J_{t}=\frac{{\left(\sum_{i=1}^{N}E_{i,t}\right)}^{2}}{N\sum_{i=1}^{N}E_{i,t}^{2}},\;J_{t}\;\in [0,1),$$

The smoothed harvested voltage is computed using an exponentially weighted moving average (EWMA), given by Equation (24):

(24) $${\bar{v}}_{t}=\left(1-\beta_{MA}\right){\bar{v}}_{t-1}+\beta_{MA}\cdot \frac{1}{N}\sum_{i=1}^{N}v_{i,t},\;$$

with $\beta_{MA}$ being the forgetting factor that controls how quickly the smoothed estimate adapts to new measurements.

#### 4.1.2. Action Space A

The action space represents the set of beamforming decisions available to the agent $a_{t}\in A$. At each time step, the agent selects a beamforming direction from a predefined angular codebook or chooses an idle (no transmission) action, given by Equation (25):

(25) $$A=\left\{\theta^{\left(1\right)},\ldots ,\theta^{\left(M\right)}\right\},\cup \left\{\emptyset \right\}\;,\;$$

Thus, $a_{t}\in A$ corresponds to either steering the beam toward one of the quantized angular positions or remaining idle, balancing energy delivery against regulatory and safety considerations.

#### 4.1.3. Reward Function R

**Figure 2:**
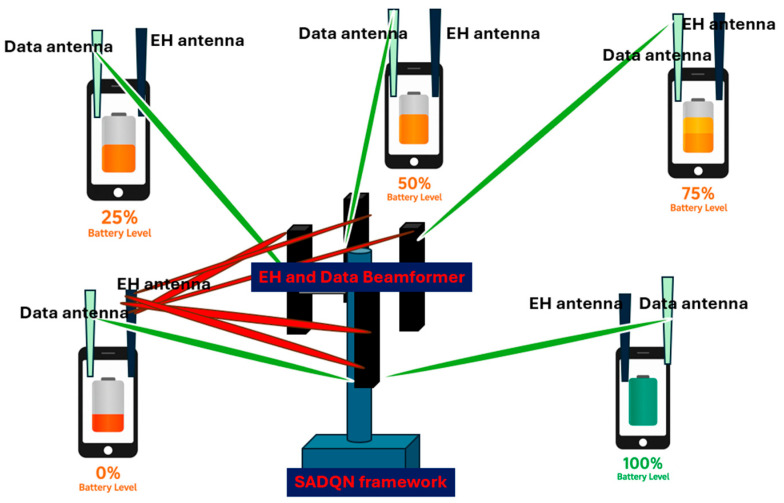
Sequential and intelligent charging reward.

The reward function R quantifies the immediate benefit of an action. Unlike conventional formulations that average energy across nodes, our framework uses a sequential charging strategy, as depicted in Figure 2, where the beamformer targets the weakest node until it reaches a survival threshold before moving to the next.

The weakest node at time t is defined as

(26) $$i^{*}\left(t\right)=arg\;\underset{i}{\min}E_{t,\;}\;$$

where the residual energy is modeled as Equation (27):

(27) $$E_{i,t}=\frac{1}{2}CV_{i,t}^{2}\;,\;$$

with C being the capacitance of the node’s energy storage element and ${\;V}_{i,t\;}$ its voltage at time t. The reward combines incremental energy improvement, alignment bonuses, spillover penalties, and survival shaping, stated in Equation (28):

(28) $${\;r}_{t}\left(s_{t},a\right)=\frac{E_{i*,t+1}-E_{i*,t}}{E_{max}}{+\;\alpha }_{align}G_{i}\left(\theta \left(a\right)\right)-\alpha_{spill}\frac{\sum_{i\neq j}P_{j,t}^{dc}}{P_{i*,t}^{dc}+\varepsilon^{\prime }}+\alpha_{surv}1\left\{V_{i*,t+1}>V_{thr}\right\},$$

- The first term normalizes the weakest node’s energy increment by $\;E_{max}$.
- The second adds an alignment bonus proportional to beamforming gain.
- The third applies a spillover penalty discouraging unnecessary energy delivery to non-target nodes.
- The final term provides a survival bonus when the weakest node surpasses the critical threshold $V_{thr}$.

#### 4.1.4. State Transition Probability Function P

The transition probability function $P\left(s_{t+1}|s_{t},a_{t}\right)$ captures how the system evolves under a given action. It reflects environmental dynamics including RF channel fading, obstacle-induced shadowing, node mobility, capacitor storage leakage, and beam steering effects. Due to the non-stationarity and partial observability of underground tunnels, these dynamics are generally unknown and are best approximated by stochastic models.

#### 4.1.5. Cost C and Budget Functions B

The CMDP incorporates multiple cost C functions, each penalizing violations of safety or operational requirements, as in Equation (29):

(29) $$C\left(s,a\right)=\left(c_{sw}\left(s,a\right),c_{fair}\left(s,a\right),c_{min}\left(s,a\right),c_{UL}\left(s,a\right)\;\right),\;$$

Each cost is paired with a strict budget B that sets the maximum allowable violation level. Table 3 summarizes the constraint definitions, and the mathematical formulations are as follows:

- Beam-Switching Constraint in Equation (30):

(30) $${\;c}_{sw,t}=1\left\{\left|\theta_{t}-\theta_{t-1}\right|>∆\theta_{thr}\right\},\;{\;b}_{sw}=0,$$

This constraint penalizes abrupt beam direction changes that exceed the threshold $∆\theta_{thr},$ ensuring smooth beam transitions that prevent destabilization of the energy-harvesting process.

ii. Fairness Constraint in Equation (31):

(31) $$c_{fair,t}={\left[\delta_{fair}-J_{t}\right]}_{+},\;b_{fair}=0,\;$$

${\left[.\right]}_{+}=max\left(0,.\right)$ denotes the positive part operator. This constraint ensures that the Jain fairness index $J_{t}$ remains above the target threshold $\delta_{fair}$, promoting equitable energy distribution across nodes.

iii. Minimum Energy Constraint in Equation (32):

(32) $$c_{min,t}=\frac{1}{N}\sum_{i=1}^{N}{\left[E_{min}-E_{i,t}\right]}_{+},\;b_{min}=0,$$

This constraint penalizes scenarios where any node’s energy falls below the minimum survival threshold $\;E_{min}$, ensuring that all nodes maintain sufficient energy for critical operations.

iv. Duty-Cycle Constraint in Equation (33):

(33) $$c_{duty,t}={\left[{\bar{u}}_{t}-\beta_{TX}\right]}_{+}\;b_{duty}=0\;,\;$$

with ${\bar{u}}_{t}$ the averaged fraction of the active slots, defined mathematically as in Equation (34):

(34) $${\bar{u}}_{t}=\frac{1}{W}\sum_{\tau =t-W+1}^{N}u_{\tau },u_{\tau }=\;\in \left\{a_{\tau }\neq \emptyset \right\},\;$$

where $u_{t}$ is a binary transmission activity variable. W is the sliding window size. This ensures compliance with regulatory duty-cycle limits $\beta_{TX}$.

v. Uplink Constraint in Equations (35) and (36):

(35) $$c_{UL,t}=\frac{1}{N}\sum_{i=1}^{N}{\left[{\bar{z}}_{i,t}-\beta_{UL}\right]}_{+,\;}\;b_{UL}=0\;,\;$$

where

(36) $${\bar{z}}_{i,t}=\frac{1}{W_{UL}}\sum_{\tau =t-W_{UL}+1}^{t}z_{i,\tau }\;,\;{\;z}_{t}\sim Bernoulli\left(\rho_{UL}\cdot 1\left\{v_{i,t}\geq V_{thr}\right\}\right),\;$$

This constraint prevents excessive uplink feedback traffic that could congest the LoRa communication channel.

#### 4.1.6. Discount Factor γ

The discount factor γ∈[0,1] controls the trade-off between immediate and long-term returns. In the EB problem, γ balances short-term voltage gains against long-term objectives such as fairness, survival, and stability of the LoRa-EH network. A typical value of γ=0.95 prioritizes long-term network sustainability while remaining responsive to immediate energy needs.

### 4.2. DQN Function Formulation

The DQN is a reinforcement learning algorithm that integrates the classical Q-learning framework with deep neural networks as function approximators. Its objective is to learn an optimal policy that maximizes the long-term cumulative reward within the CMDP formulation of our problem. In this work, DQN provides the computational foundation for adaptive decision-making in energy beamforming under underground RF-WPT constraints. Formally, the optimal state–action value function $Q^{*}\left(s_{t},a\right)$ is defined recursively by the Bellman optimality equation [22] in Equation (37):

(37) $${\;Q}^{*}\left(s_{t},a\right)=r\left(s_{t},a\right)+\gamma \sum_{s^{\prime }}P\left(s_{t+1}|s_{t},a\right)\underset{a^{\prime }}{\max}\;Q^{*}\left(s_{t}^{\prime },a^{\prime }\right),\;$$

which expresses the expected utility of taking action a in state $s_{t}$, accounting for both immediate reward and discounted future returns. In DQN, the state action value function is approximated using a deep neural network parameterized by ψ in Equation (38):

(38) $${\;Q}_{\psi }\left(s_{t},a\right)\approx Q^{*}\left(s_{t},a\right),\;$$

where ψ represents the set of trainable weights and biases. The parameters are optimized by minimizing the Bellman loss in Equation (39):

(39) $$L\left(\psi \right)=E_{\left(s_{t},a,r_{t},s_{t+1}\right)}\left[{\left(Y_{t}-Q_{\psi }\left(s_{t},a\right)\right)}^{2}\right],\;$$

with the target value defined as in Equation (40):

(40) $${\;Y}_{t}=r_{t}+\;\gamma \;\underset{a^{\prime }}{\max}Q_{\psi -}\left(s_{t+1},a^{\prime }\right)\;,\;$$

where ψ are the parameters of a target network updated periodically to stabilize learning. The Double DQN (DDQN) extends this formulation by decoupling the action selection and action evaluation steps, thereby reducing overestimation bias. More broadly, DRL follows a trial-and-error learning paradigm, where the agent interacts with the environment, accumulates experience, and iteratively refines its policy. In the context of underground WPT, the agent must learn an adaptive beamforming policy conditioned on state information $s_{t}$, which encodes harvested node voltages, fairness indicators, and the prior beam direction $\theta_{t-1}$. The DQN evaluates the expected long-term utility of steering the beam toward candidate directions θ(a). A fundamental challenge lies in balancing **exploration**, i.e., testing new beam directions to improve environmental knowledge, with **exploitation,** choosing actions known to maximize energy transfer efficiency. This balance is particularly critical in dynamic, safety-critical underground networks, where channel fading, obstructions, and node mobility demand robust and adaptive beam-steering strategies.

### 4.3. Problem Formulation

The Safe DQN extends the conventional DQN framework by optimizing the expected return while explicitly enforcing one or more operational constraints, $c_{i}\in C$. The objective is to derive a policy π that maximizes the discounted harvested energy reward while adhering to safety requirements such as beam-switching stability, fairness, minimum energy guarantees, and uplink usage limits. Formally, the primal problem is defined as Equation (41):

(41)  (P0)max πEπ[∑t=0∞γtrt] ,

subject to

$$E_{\pi }\left[c_{j,t}\right]\;\leq b_{j}\;j\;\in \left(c_{sw,t},c_{fair,t},c_{min,t},c_{UL,t}\;\right)$$

where $c_{j,t}$ denotes the constraint costs at time t, and $b_{j}$ are the corresponding budget thresholds, as defined previously in (30)–(36). These constraints ensure that the learned policy maintains operational safety, fairness across nodes, and compliance with system-level limitations, which are critical in underground RF-WPT environments. In the literature, the standard approach to solving CMDPs is to transform the constrained optimization problem into an unconstrained one using the Lagrangian relaxation method. Specifically, (P0) is referred to as the primal problem, and by introducing non-negative Lagrange multipliers $\lambda_{j}\geq 0$ associated with each constraint, one can formulate the dual problem. This transformation enables the use of gradient-based learning to simultaneously update both the policy and the multipliers, ensuring that energy efficiency is maximized while constraint violations are adaptively penalized. The detailed solution approach is presented in Section 5.

## 5. Proposed EB Optimization Solution

Given the harsh RF propagation conditions in underground mines, our objective is to maximize real-time WPT efficiency while ensuring quality-of-service (QoS) constraints are satisfied. The optimization problem (P0) formulated in Section 4 is inherently nonlinear due to the structure of the constraint functions and is computationally intractable (NP-hard) to solve directly. This section presents our solution approach based on Lagrangian relaxation and dual optimization.

### 5.1. Safe-DQN Lagrangian-Based Solution

To address the NP-hard constrained optimization problem, we decompose the solution into three key steps: (i) Lagrangian relaxation to transform the constrained problem into an unconstrained one, (ii) dual optimization to solve the relaxed problem, and (iii) Lagrangian-based Q-function formulation for practical implementation with Deep Q-Networks.

#### 5.1.1. Lagrangian Relaxation

The Lagrangian relaxation technique transforms the CMDP into an unconstrained optimization problem by introducing non-negative Lagrange multipliers $\lambda_{j}\geq 0$ for each constraint function $c_{j,t}.$ The discounted-trajectory Lagrangian is expressed as Equation (42):

(42) $$L\left(\pi ,\lambda \right)=E_{\pi }\left[\sum_{t=0}^{\infty }\gamma^{t}\left(r_{t}-\sum_{j}\lambda_{j}c_{j,t}\right)\right]+\sum_{j}\lambda_{j}b_{j}\;,$$

Equation (42) reformulates the Safe-DQN CMDP into a single-objective optimization problem where violations of safety constraints are penalized by the multipliers $\lambda_{j}$. The key insight is that when a constraint is violated, i.e., $\left(c_{j,t}>b_{j}\right)$, the term $-\lambda_{j}c_{j,t}$ reduces the reward, discouraging the agent from selecting actions that lead to constraint violations. Conversely, when constraints are satisfied, i.e., $\left(c_{j,t}<b_{j}\right)$, the penalty is minimal or zero, allowing the agent to focus on maximizing energy harvesting. For computational efficiency, we define a shaped per-step reward that embeds these penalties directly as Equation (43):

(43) $${\bar{r}}_{t}\left(\lambda \right)=r_{t}-\sum_{j\in \left\{c_{sw}c_{fair},c_{min},c_{UL}\right\}}\;\lambda_{j}c_{j,t,\;}$$

With this formulation, the agent can treat the CMDP as a standard MDP with adjusted rewards that automatically incorporate safety considerations. This eliminates the need to explicitly check constraints at each step, simplifying the learning process while maintaining safety guarantees.

#### 5.1.2. Dual Optimization

The Lagrangian relaxation leads to a saddle-point problem, defined as Equation (44):

(44) $$\underset{\lambda \geq 0}{\mathrm{Min}\;}\underset{\pi }{\max}L\left(\pi ,\lambda \right),\;$$

A saddle point represents an equilibrium where

- The primal update (inner maximization) adjusts the policy π to maximize the penalized reward.
- The dual update (outer minimization) adjusts the multipliers’ λ penalize constraint violations.

Formally, a pair $\left(\pi^{*},\lambda^{*}\right)$ is a saddle point if Equation (45) holds:

(45) $$L\left(\pi^{*},\lambda \right)\leq L\left(\pi^{*},\lambda^{*}\right)\leq L\left(\pi ,\lambda^{*}\right)\;\forall_{\pi },\lambda \geq 0,$$

##### Convergence Guarantee

The optimality of $\left(\pi^{*},\lambda^{*}\right)$ is characterized using the Karush–Kuhn–Tucker (KKT) conditions, which must hold simultaneously at equilibrium:

- *Primal Feasibility* (constraints satisfied) as Equation (46):

(46) $$E_{\pi }\left[c_{j,t}\right]\leq b_{j},{\;\forall }_{j},\;$$

This ensures that the learned policy respects all safety constraints on average.

ii. *Dual Feasibility* (multipliers nonnegative) as Equation (47):

(47) $${\;\lambda }_{j}\geq 0,{\;\forall }_{j},\;$$

Non-negative multipliers ensure that constraint violations are always penalized, never rewarded.

iii. *Stationarity* (optimal policy maximizes the Lagrangian for fixed multipliers) as Equation (48):

(48) $$\pi^{*}=\arg\underset{\pi }{\max}L\left(\pi ,\lambda^{*}\right),\;$$

At equilibrium, no policy can achieve higher penalized reward than $\pi^{*}$ given the multipliers $\lambda^{*}$.

iv. *Complementary Slackness* (constraints only “bind” if they are tight) as Equation (49):

(49) $$\lambda_{j}^{*}\left(E_{\pi }\left[c_{j,t}-b_{j}\right]\right)=0\;\forall_{j},\;$$

If a constraint is not violated, i.e., $E_{\pi }\left[c_{j,t}\right]<b_{j},$ then, $\lambda_{j}^{*}=0$ and no penalty is needed. If a constraint is tight, i.e., $E_{\pi }\left[c_{j,t}\right]>b_{j}$, then $\lambda_{j}^{*}>0$ and a penalty is active. This adaptive penalty mechanism ensures efficient constraint enforcement. At equilibrium, these KKT conditions guarantee that the algorithm maximizes long-term WPT efficiency while strictly satisfying all operational and safety constraints in underground RF-WPT environments.

#### 5.1.3. Lagrangian-Based Q-Function

Finally, the principle of Bellman optimality can be extended to incorporate Lagrangian penalties. The Lagrangian-based Q-function is defined as Equation (50):

(50) $$Q_{\lambda }\left(s_{t},a\right)=E_{\pi }\left[{\bar{r}}_{t}\left(s_{t},a\right)-\underset{a^{\prime }}{\mathrm{\gamma max}}Q_{\lambda }\left(s_{t+1},a^{\prime }\right)\right]\;,\;$$

$a^{\prime }$ denotes the next action under the principle of optimality, and ${\bar{r}}_{t}$ is the shaped reward from Equation (43). Equation (50) implies that the agent learns $Q_{\lambda }\left(s_{t},a\right)$ to always select the action that yields

- Energy-harvesting gains (positive contribution from $r_{t}$).
- Constraint violation penalties (negative contribution from $\sum_{j}\lambda_{j}c_{j,t}$).

Thus, the proposed Safe Adaptive DQN algorithm alternates between

- Primal policy updates: Maximizing shaped rewards ${\bar{r}}_{t}$ (maximizing energy while respecting penalties).
- Dual updates: Adjusting multipliers $\lambda_{j}$ (increasing penalties when constraints are violated).

This iterative process ensures convergence to a policy that balances energy efficiency with strict adherence to QoS and safety constraints in underground RF-WPT environments.

### 5.2. Adaptive Mechanisms in Safe-DQN Beamforming

Thus far, the Safe-DQN framework has been described in terms of its fundamental reinforcement learning and constraint-handling principles. We now introduce the adaptive mechanisms that extend the baseline Safe-DQN agent beyond conventional reward maximization, enabling robust and resilient energy beamforming in underground RF-WPT networks. The proposed Safe Adaptive DQN (SADQN) integrates five key components:

- Energy-Aware (EA) score that prioritizes the most energy-starved node.
- Fairness Bonus (FB) that incentivizes a balanced energy distribution across all nodes.
- Adaptive Exploration that dynamically tunes the exploration probability based on system conditions.
- Composite Score to rank beamforming actions.
- Fairness-Aware *Top-K* action selection that prevents overly greedy or myopic behavior.

These mechanisms are fused into a composite score function, which ranks candidate beamforming actions by combining Q-values with adaptive penalties and incentives. The following subsections define and discuss each mechanism in detail.

#### 5.2.1. Energy-Aware (EA)

The energy-aware score biases the agent toward nodes with critically low energy reserves, implementing a triage principle essential for disaster scenarios. The harvested energy increment for the weakest node under the beamforming direction θ(a) is normalized to obtain Equation (51):

(51) $$EA\left(\theta \left(a\right)|s_{t}\right)=\frac{E_{i,t+1}^{*}|\left(a\right)-E_{i,t}^{*}}{E_{max}},\;$$

where $E_{i,t+1}^{*}$ denotes the predicted residual energy of the weakest node (identified via Equation (26)) after taking action a, and $E_{max}$ is the maximum storable energy. By emphasizing this score, the agent allocates beams that extend the operational lifetime of the most vulnerable nodes, an essential feature for disaster-resilient underground networks.

#### 5.2.2. Fairness Bonus (FB)

To complement the hard fairness constraint $c_{fair,t}$, we introduce a fairness bonus as a soft incentive. This term evaluates how an action improves the energy balance across all nodes, leveraging Jain’s fairness index (Equation (23)), as Equation (52):

(52) $$FB\left(\theta \left(a\right)|s_{t}\right)=J\left(V_{i,t+1}|\left(a\right)\right)\;,\;$$

J∈[0,1] measures the fairness of the post-decision energy distribution, with J→1 indicating perfect fairness (all nodes have equal energy) and J→0 indicating extreme inequality. This encourages the agent to pursue actions that enhance equity across nodes, even when fairness constraints are not immediately binding.

#### 5.2.3. Adaptive Exploration

Unlike conventional DQN, which employs a fixed or monotonic ε-decay schedule, our approach adjusts exploration dynamically in response to system variability. The key insight is

- When harvested voltages across nodes exhibit high dispersion (unbalanced system), increase exploration to discover better beam directions.
- When the system is stable and balanced, prioritize exploitation of known good actions.

The adaptive exploration rate is defined as Equation (53):

(53) $$\varepsilon_{t}=clip\left(\varepsilon_{min}+\alpha_{\varepsilon }\frac{std\left(v_{i,t}\right)}{V_{max}},\varepsilon_{min},\varepsilon_{max}\right),\;$$

- $std\left(v_{i,t}\right)$ is the standard deviation of harvested voltages across nodes.
- $\alpha_{\varepsilon }$ is a scaling factor.
- $\varepsilon_{min},\varepsilon_{max}$ are exploration bounds.

This mechanism enables the agent to adapt exploration rates to network conditions, improving responsiveness in highly dynamic underground environments.

#### 5.2.4. Composite Score

The adaptive mechanisms are integrated into a composite score used to rank beamforming actions, defined as Equation (54):

(54) $$S\left(s_{t},a\right)=Q_{\psi }\left(s_{t},a\right)-\sum_{J}\lambda_{J}c_{i,j}\left(s_{t},a\right)+\eta_{prio}\cdot EA\left(a\right)-\;\eta_{sw}\cdot \psi_{sw}\left(a\right)+\;\mu \cdot FB\left(a\right),\;$$

- $Q_{\psi }\left(s_{t},a\right)$: Q-value predicted by the neural network (expected long-term return).
$-\sum_{J}\lambda_{J}c_{i,j}\left(s_{t},a\right)$: Constraint penalties from Lagrangian formulation.
- $+\eta_{prio}\cdot EA\left(a\right)$: Bonus for helping the weakest node (triage principle).
- $\eta_{sw}\cdot \psi_{sw}\left(a\right)\;$: Penalizes abrupt beam switches through the switching indicator $\psi_{sw}\left(a\right)=1\left\{a\neq \emptyset \;\Lambda \left|\theta \left(a\right)-\theta_{t-1}\right|>{△\theta }_{thr}\right\}$.
- μ·FB(a): Bonus for improving fairness.

This composite score balances multiple objectives:

- High score → good action (high energy, low violations, helps weak nodes, fair, smooth).
- Low score → poor action (low energy, many violations, ignores weak nodes, unfair, erratic).

The weighting parameters $\eta_{prio}$, $\eta_{sw}$, μ allow for tuning the relative importance of each objective.

#### 5.2.5. Fairness-Aware Top-K Selection

To avoid myopic selection of a single argmax action, we adopt a Fairness-Aware Top-K mechanism. At each decision step, a subset of high-ranking candidate actions is retained: selects a subset of strong candidate actions defined as Equation (55):

(55) $$A_{t}^{\left(k\right)}=Top\;k_{a}\left\{S\left(s_{t},a\right)\right\}\;,\;$$

where adaptive $k_{t}$ is defined as

(56) $${\;k}_{t}=k_{min}+\left\lfloor \left(k_{max}-k_{min}\right)\cdot \frac{std\left({\;E}_{t}\right)}{{\;E}_{max}}\right\rfloor \;,$$

The final action is then selected from this candidate set according to Equation (57):

(57) $${\;a}_{t}=\{\begin{array}{c} Uniform\;\left(A_{k}^{\left(t\right)}\right),\;with\;prob\cdot \;{\;\varepsilon }_{t}\;\\ arg\;\underset{a\in A_{k}^{\left(t\right)}}{\max}s\left(a\right),\;with\;prob\cdot \;1-{\;\varepsilon }_{t}\; \end{array},\;$$

The idle action ∅ is always included to ensure compliance with duty-cycle constraints. Intuitively, when fairness is low, $k_{t}$ is small and exploration is restricted to a few high-priority beams, while high fairness allows larger $k_{t}$, enabling broader exploitation of good beamforming directions.

### 5.3. SADQN Energy Beamforming Algorithm

We propose the Safe Adaptive Deep Q-Network (SADQN) algorithm to address the dynamic and safety-critical nature of underground mining disaster environments. Unlike plain DQN or self-DQN, the SADQN framework introduces adaptive mechanisms that adjust exploration in response to channel variability while strictly enforcing operational constraints. This ensures that the algorithm not only maximizes harvested energy but also maintains fairness, safety, and reliability under extreme RF conditions.

In practice, SADQN balances two competing goals: (i) maximizing received RF power through adaptive beam steering, and (ii) satisfying operational constraints, such as transmit duty cycle, fairness across nodes, and beam-switching stability. This makes SADQN particularly suitable for safety-critical underground networks, where reliable energy provisioning to LoRa-enabled nodes is essential for localization, sensing, and emergency communications. Algorithm 1 presents the proposed SADQN EB optimization algorithm.

**Algorithm 1** Adaptive Safe-DQN-Based Energy Beamforming Algorithm***Inputs:***Angle codebook: A= $\left\{\theta^{\left(1\right)},\ldots ,\theta^{\left(n\right)}\right\},$ plus idle action ∅Discount: γ∈[0,1]; Replay capacity: C; Batch size: B.Target update (soft): $\tau_{update}\in (0,1]$, EWMA factor: $\beta_{MA}$.Constraint targets: $\delta_{fair}$, $E_{min},\;\beta_{TX}$, $\beta_{UL}$.Exploration: $\left(\varepsilon_{0},\varepsilon_{min},\;\delta_{\varepsilon }\right)$ or adaptive $\varepsilon_{t}$.Switching threshold ${△\theta }_{thr},$ dual step: $\eta_{\lambda }$.Top-*K* bounds: ($k_{min},\;k_{max}$).Priority/fairness/switch weights: $\eta_{prio}$ (bias toward low-energy node), $\;\eta_{sw}$ (penalty for large abrupt beam switches), μ (fairness bonus).**Initialization**1:Initialize Q-network $Q_{\psi }\left(s_{t},a\right)$ with random weights; set target network $Q_{\psi -}\leftarrow \;Q_{\psi }$2:Create replay buffer D of capacity C3:Initialize duals $\lambda_{sw}=\lambda_{fair}=\lambda_{min}=\lambda_{duty}=\lambda_{UL}=0$4:Initialize parameters $\varepsilon \leftarrow \varepsilon_{0},\;\theta_{prev}\leftarrow 0,\bar{v}\leftarrow 0,\bar{J}\leftarrow 0$5:Initialize sliding duty trackers: $\bar{u}$, $\left\{{\bar{z}}_{i}\right\}$, $\left\{E_{i}\right\}$6:Loop7:**for** each episode: **do**8:     Reset environment if episodic9:     **for** each time step t: **do**10:      **State construction**11:      Set $s_{t}=\left[\bar{v}/V_{max},\left(\theta_{t-1}+\frac{\pi }{2}\right)/\pi ,\bar{J}\right]$: Equation (22)   Optionally append $\bar{u},$ and $\bar{z}$ pooled statistic12:   **Energy-Aware Scoring**13:    Select target by residual energy: Equation (26)14:     **for** each α∈A∪{∅} : **do**15:     predict one-step next energies $E_{i,t+1}|\left(a\right)$ Equation (16) predict voltages     $V_{i,t+1}|\left(a\right)$ Equation (17)16:      Set switch flag    $\psi_{sw}\left(a\right)=1\left\{a\neq \emptyset \;\Lambda \left|\theta \left(a\right)-\;\theta_{t-1}\right|>\;{△\theta }_{thr}\right\}$17:    Compute: EA(a): Equation (51), FB(a): Equation (52) and Composite scoring function      $S\left(s_{t},a\right)$ (Equation (54)) including $\eta_{prio}$, $\eta_{sw}\;$, μ18:      **End for**19:  **Fairness-Aware Top-*K***20:   Compute adaptive $k_{t};\;$ Equation (56) and form candidate set $A_{t}^{\left(k\right)};$ Equation (55)21:  **Action Selection**22:   With probability $\varepsilon_{t}\;$ (adaptive) or ε (fixed) sample funiformly from $\left(A_{k}^{\left(t\right)}\right)\;$23:   Otherwise $a_{t}=\;arg\;\underset{a\in A_{k}^{\left(t\right)}}{\max\;}s\left(a\right)$; Equation (57)24: **Environment Update**25: Execute $a_{t};$ set $u_{t}=1$ **if** $u_{t}\neq \;\emptyset$ **else** $u_{t}=0$26: Observe voltages $\left\{v_{i,t}\right\};$ update node energies; Equation (16)27: Compute reward $r_{t}$; Equation (28)28: Update trackers $\bar{u},\;\left\{{\bar{z}}_{i}\right\},\bar{v},\;\bar{J}$ via EWMA factor $\beta_{MA}$; Equation (24)29: **Constraints Costs**30: Compute $c_{sw,t},c_{fair,t},c_{min,t},c_{duty,t},c_{UL,t}$; Equations (30)–(36)31: **Shaped Rewards and Storage**32: Set ${\bar{r}}_{t}=r_{t}-\sum_{j}\lambda_{j}c_{j,t}$; Equation (40)33: Push $\left(s_{t},a_{t},\;{{\bar{r}}_{t},s_{t+1},d}_{t}\right)$ into D34: Learning (Double-DQN)35: **if**      |D|≥B **Then**36: Sample a mini-batch from D37: **for** each sample, compute target; Equation (41)38:  $a^{*}=arg\;\underset{a^{\prime }}{\max\;}Q_{\psi },\left(s_{t+1},a^{\prime }\right)$39:   $Y_{t}={\bar{r}}_{t}\;+\;\gamma \left(1-d_{t}\right)Q_{\psi -}\left(s_{t+1},a^{*}\right)$40:   Update ψ by minimizing loss ${L=\left(Q_{\psi }\left(s_{t},a\right)-Y\right)}^{2}$41:  **End if**42: Soft update (every step)43:  $Q_{\psi -}\leftarrow {\tau_{update}*Q}_{\psi }+\left(1-\tau_{update}\right)*Q_{\psi -}$44: Dual Updates45: **for** each constraint *j*: **do**46:  $\lambda_{j}\;\leftarrow max\left(0,\lambda_{j}+\eta_{\lambda }\left(c_{j,t}-b_{j}\right)\right)$47:  **End for**48:Bookkeeping49:Update $\varepsilon \leftarrow max\left(\varepsilon_{min},\;\varepsilon \cdot \delta_{\varepsilon }\right)\;$ if using fixed decay50:**if**$a_{t}\neq \emptyset$ **then,** update $\theta_{prev}=\theta \left(a_{t}\right)$51: **End for** (time steps)52: **End for (Episodes)**
  Output
The algorithm yields a trained Q-network $Q_{\psi }$ and optimized multipliers $\left\{\lambda_{j}\right\}.$ The final deployment policy $\pi^{*}{=argmax}_{a}Q_{\psi }\left(s,a\right)$ augmented with Energy-Aware Top-*K* gating mechanism.

## 6. Simulations and Analysis

In this section, we present a comprehensive performance evaluation of the proposed Safe Adaptive Deep Q-Network (SADQN)-based energy beamforming framework through extensive simulations. To rigorously assess the effectiveness of our approach, we compare SADQN against a diverse set of baseline algorithms spanning reinforcement learning methods, classical optimization techniques, and analytical benchmarks. This comparative analysis aims to demonstrate the advantages of incorporating safety-aware learning mechanisms with adaptive exploration in underground wireless power transfer scenarios while quantifying the inherent trade-offs between energy maximization, constraint satisfaction, and system fairness.

### 6.1. Baseline Algorithms

The performance of SADQN is evaluated against the following eight baseline algorithms, each representing a distinct approach to the energy beamforming problem. Our algorithms were compared with the following baseline algorithms: (i) Deep Q-Network (DQN): The standard value-based deep reinforcement learning algorithm that optimizes beamforming decisions without explicit safety considerations, (ii) Safe-DQN: Our constrained reinforcement learning framework that integrates Lagrangian-based constraint handling into the DQN architecture through a dual variable, (iii) Deep Deterministic Policy Gradient (DDPG): An actor–critic reinforcement learning algorithm capable of handling continuous action spaces, (iv) Particle Swarm Optimization (PSO): A population-based metaheuristic optimization algorithm inspired by social behavior of bird flocking, (v) Random Beamforming: A naive policy that randomly selects beam directions from the angular codebook with uniform probability at each time step, without learning or optimization, and (vi) Non-Beamforming (NB): An omnidirectional transmission strategy where the transmitter radiates equally in all directions without spatial selectivity.

### 6.2. Performance Metrics

The algorithm performance is evaluated across five key categories critical for disaster-tolerant underground mining networks: (i) Energy-harvesting Metrics, (ii) Safety and Constraint Metrics, (iii) Fairness Metrics, (iv) Operational Metrics, and (v) Robustness Metrics.

### 6.3. Simulation Environment and Parameters

#### 6.3.1. Underground Mine Channel Model

Simulations employ a realistic underground mine propagation model incorporating waveguide effects, multipath fading, shadowing, and moisture absorption as detailed in Section 3.2. The channel model combines

- Path loss with corridor propagation exponent $n_{Corridor}=2.2$.
- Rician fading with K-factor = 5 dB modeling LoS and scattered components.
- Log-normal shadowing with standard deviation σ = 6 dB.
- Wall penetration loss $L_{Wall}=25$ dB.
- Junction scattering loss $L_{Junction}=8$ dB.
- Moisture absorption $L_{Moist}=2–5$ dB.

Node mobility follows a Gauss–Markov random walk model with temporal correlation coefficient $\alpha_{0}=0.8$ and angular drift variance ${\;\sigma }_{drift}=0.002$ radians, emulating realistic miner and equipment movement patterns in underground tunnels.

#### 6.3.2. System Configuration

**Table 4:** Simulation parameters.

Parameter	Symbol	Value	Unit
**RF-WPT Transmitter**			
Carrier frequency	$f_{c}$	860	-
Wavelength	$\lambda_{0}$	0.349	m
Transmit power	$P_{T}$	30	dBm
Number of antenna elements	$N_{t}$	16	-
Antenna gain per element	$G_{elem}$	3	dBi
Inter-element spacing	d	$0.5\;\lambda_{0}$	m
Angular span	$\theta_{span}$	120°	degrees
Angular codebook size	‖A‖	48	beams
**LoRa EH Nodes**			
Number of nodes	N	4	-
LoRa uplink frequency	$f_{LoRa}$	915	MHz
Energy storage capacitance	C	1.0	Farad
Minimum stored energy	$E_{min}$	0.5	J
Maximum stored energy	$E_{max}$	5.0	J
Load power consumption	$P_{lοad}$	2	mW
RF-to-DC conversion efficiency	$\eta_{RF-DC}$	0.6	
Leakage time constant	$\rho_{leak}$	1000	s
**Channel and Environment**			
Rician K-factor	K	5	dB
Shadowing std. deviation	$\sigma_{Shadow}$	6.0	dB
Reference path loss	$PL\left(d_{0}\right)$	40	dB
Corridor propagation exponent	$n_{Corridor}$	2.2	
Node maximum speed	$\omega_{max}$	0.5	m/s
AoA drift variance	${\;\sigma }_{drift}$	0.002	rad
Waypoint tolerance	${∄}_{wp}$	0.3	m
**Operational Constraints**			
Maximum duty cycle	$\beta_{TX}$,	0.40	
Fairness threshold	$\delta_{fair}$	0.85	
Minimum energy threshold	$E_{min}$	0.5	J
Maximum uplink utilization	$\beta_{UL}$	0.30	
Smooth switching threshold	${△\theta }_{thr}$	20°	degrees
Time slot duration	τ	0.5	s
**DQN Hyperparameters**			
Discount factor	γ	0.95	
Learning rate	α	0.001	
Replay buffer size	‖D‖	10,000	transitions
Batch size	B	64	samples
Initial exploration rate	$\varepsilon_{0}$	1.0	-
Minimum exploration rate	$\varepsilon_{min}$	0.05	-
Exploration decay factor	$\delta_{\varepsilon }$	0.995	-
Target network update rate	$\tau_{update}$	0.005	-
Hidden layer dimensions	-	[128, 128]	neurons
Activation function	-	ReLU	-
**SADQN-Specific Parameters**			
Adaptive exploration scaling	$\alpha_{\varepsilon }$	0.3	-
Energy-aware weight	$\eta_{prio}$	0.5	-
Switching penalty weight	$\eta_{sw}$	0.2	-
Fairness bonus weight	μ	0.3	-
Top-K minimum	$k_{min}$	3	actions
Top-K maximum	$k_{max}$	10	actions
EWMA factor	$\beta_{MA}$	0.05	-
Dual ascent step size	$\eta_{\lambda }$	0.01	-
**DDPG Hyperparameters**			
Actor learning rate	$\alpha_{actor}$	0.0001	-
Critic learning rate	$\alpha_{critic}$	0.001	-
Soft update parameter	$\tau_{DDPG}$	0.001	-
Ornstein–Uhlenbeck noise	$\sigma_{OU}$	0.2	-
**PSO Hyperparameters**			
Swarm size	-	20	particles
Inertia weight	w	0.7	-
Cognitive coefficient	$c_{1}$	1.5	-
Social coefficient	$c_{2}$	1.5	-
**Training Configuration**			
Number of episodes	-	500	-
Steps per episode	-	100	-
Random seeds	-	10	-
Evaluation frequency	-	10	episodes
Moving average window	W	20	steps

#### 6.3.3. System Network Topology

The underground mine testbed consists of four LoRa-enabled energy-harvesting nodes deployed along a 50 m tunnel segment. Initial node positions are uniformly distributed within the tunnel, with subsequent positions evolving according to the Gauss–Markov mobility model. The transmitter is positioned at one end of the tunnel with a 16-element ULA capable of steering beams across Table 4: Simulation Parameters ±60° azimuth range, discretized into 48 beam directions (2.5° resolution).

#### 6.3.4. Experimental Scenarios

Two primary scenarios are evaluated to capture the dual challenges of underground wireless power transfer:

- **Stationary Scenario**: Nodes remain at fixed positions throughout each episode, isolating the algorithms’ ability to learn optimal beam steering under static channel conditions with only fading variability. This scenario tests steady-state convergence and exploitation efficiency.
- **Mobile Scenario**: Nodes move according to the Gauss–Markov mobility model at speeds up to 0.5 m/s, emulating realistic miner and equipment movement. Dynamic positions introduce time-varying channel conditions requiring continuous adaptation. This scenario tests the algorithms’ ability to track moving targets and maintain energy delivery under non-stationary environments.

#### 6.3.5. Evaluation Protocol

Each algorithm is trained and evaluated using the following protocol:

- Initialization: All learnable parameters are randomly initialized; replay buffers are empty; dual variables set to zero.
- Training Phase: Algorithms interact with the environment for 500 episodes (50,000 total time steps).
- Multiple Seeds: Each experiment is repeated across 10 random seeds to ensure statistical robustness.
- Performance Logging: Metrics are recorded at every time step and aggregated at episode boundaries.
- Statistical Analysis: Results are reported as mean ± standard deviation across seeds, with box plots showing the distribution.

All simulations are implemented in Python 3.9 using PyTorch 1.12 for neural network components. The complete codebase, including environment models and trained weights, will be made publicly available upon publication to ensure reproducibility.

### 6.4. Results and Discussion

The following subsections present a detailed comparative analysis organized by performance dimensions. Each subsection includes quantitative results, visual comparisons, and qualitative insights into algorithm behavior.

#### 6.4.1. Cumulative Reward

**Figure 3:**
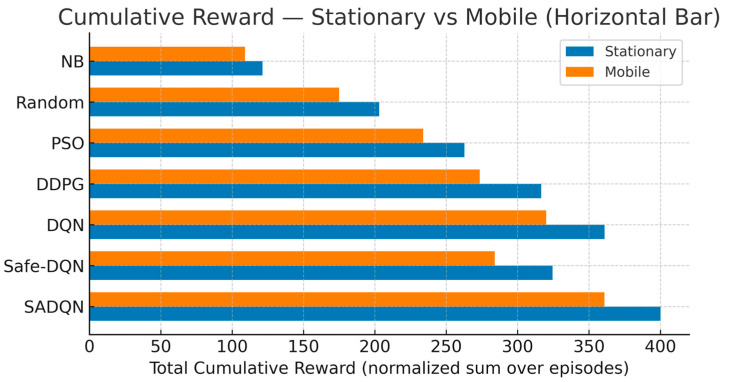
Cumulative reward function comparison.

Figure 3 compares the total cumulative rewards achieved by the proposed SADQN, Safe-DQN, and benchmark algorithms under both stationary and mobile scenarios.

The cumulative reward integrates the agent’s long-term optimization of harvested energy, fairness, and duty-cycle constraints within the constrained MDP formulation. SADQN exhibits the highest cumulative reward, validating its adaptive exploration and energy-aware scoring strategy for sustainable WPT operation. The marginal reduction in the mobile case reflects the performance trade-off introduced by temporal channel fading and node mobility dynamics. PSO can become trapped in local optima, degrade as dimensionality increases (the curse of dimensionality), and adapt poorly in changing environments. In contrast, DQN leverages deep neural networks to handle large, complex state spaces and learn near-optimal policies, which makes it more suitable for dynamic, sequential decision problems—although it typically requires more data and computational resources. PSO is comparatively simpler and can converge faster on continuous, static optimization tasks, but it generally falls short on problems with complex time-coupled decisions where deep reinforcement learning performs best.

#### 6.4.2. Duty Cycle

**Figure 4:**
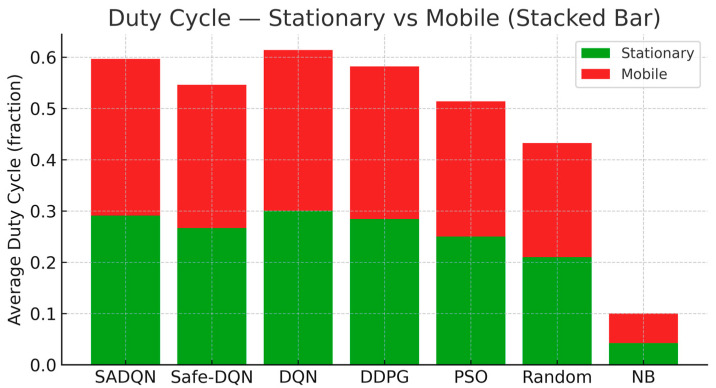
Duty-cycle utilization function comparison.

The stacked bars in Figure 4 depict average duty-cycle utilization, showing the proportion of active transmission time for stationary and mobile environments. Duty-cycle regulation ensures compliance with underground wireless standards and prevents excessive channel occupancy in disaster-recovery conditions. SADQN maintains near-optimal transmission duty levels, balancing channel access with harvested-energy fairness to maximize network longevity. The increased duty usage in mobile conditions highlights the adaptive scheduling overhead required for dynamic topology updates and link re-synchronization.

#### 6.4.3. Fairness

**Figure 5:**
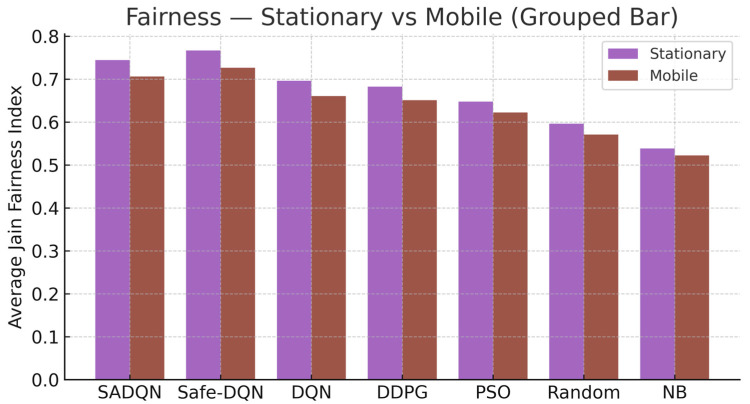
Fairness function comparison.

Figure 5 presents the mean Jain fairness index across episodes, capturing energy distribution equity among LoRa EH nodes. Fairness optimization is embedded in the SADQN reward design via the energy-aware Top-K constraint and Lagrange-penalized dual update. SADQN and Safe-DQN achieve superior fairness stability, minimizing variance in harvested energy across heterogeneous nodes. Minor fairness degradation in the mobile setting stems from spatial energy fluctuation and intermittent relay selection during motion-induced fading.

#### 6.4.4. Convergence Speed

**Figure 6:**
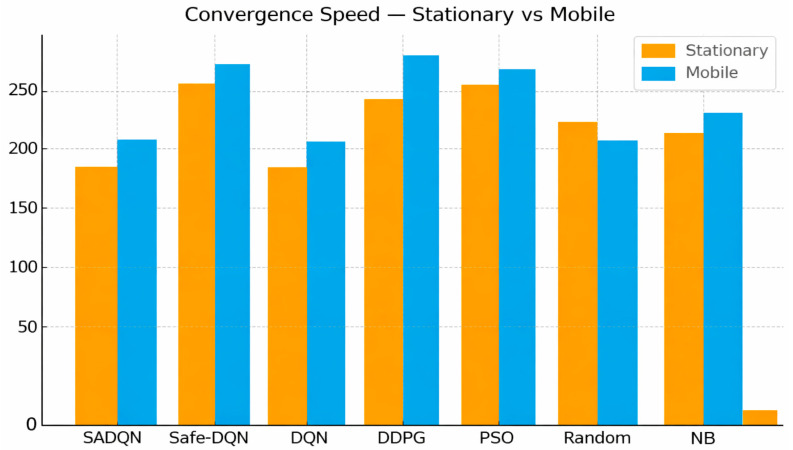
Convergence speed comparison.

Figure 6 compares the learning convergence rate of each algorithm, expressed as the number of episodes required to reach 90% of steady-state reward. SADQN converges significantly faster than Safe-DQN, DDPG, and PSO, confirming the efficiency of its adaptive exploration and energy-aware policy updates. Its rapid convergence indicates enhanced learning stability and reduced sample complexity in dynamic wireless power transfer environments. The slower convergence in mobile conditions reflects additional exploration required under time-varying channel and topology uncertainty in underground scenarios.

#### 6.4.5. Energy Efficiency

**Figure 7:**
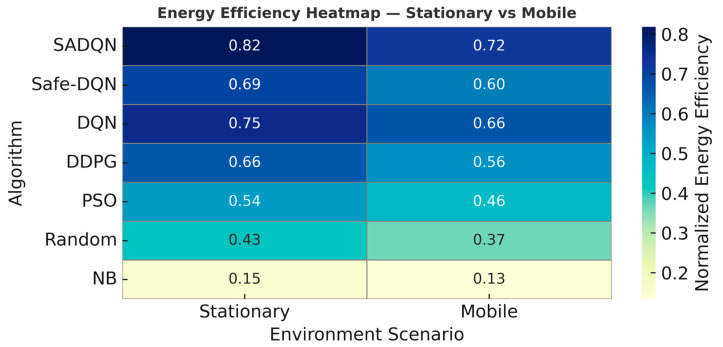
Energy efficiency across the algorithms.

The heatmap in Figure 7 visualizes the normalized energy efficiency of all algorithms under stationary and mobile network conditions. Energy efficiency quantifies the ratio of harvested-to-transmitted power achieved by each policy during long-term RF wireless power transfer. SADQN demonstrates the highest efficiency across both environments, validating its energy-aware reward design and adaptive beam selection capability. Efficiency degradation in mobile conditions highlights the impact of dynamic channel fading and positional drift, reinforcing the need for adaptive safe reinforcement learning in underground mining scenarios.

#### 6.4.6. Constraint Cost Analysis

**Figure 8:**
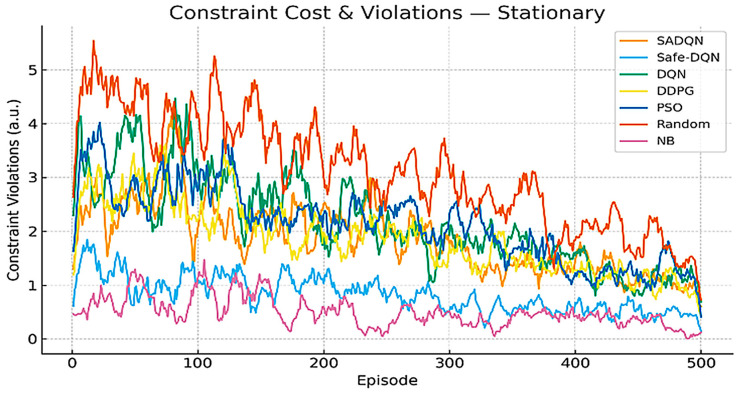
Constraint cost violation in stationary scenario.

**Figure 9:**
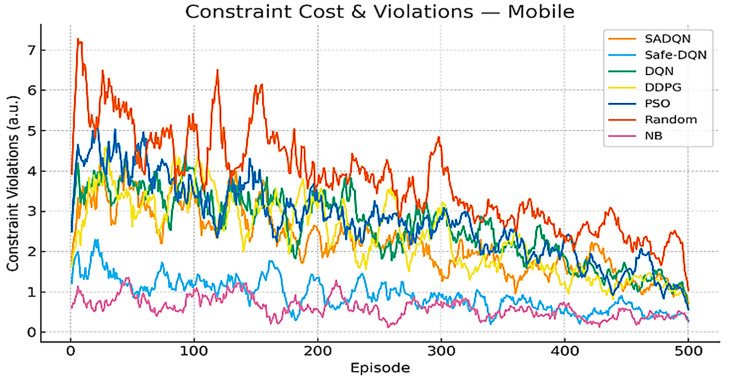
Constraint cost violation in mobile scenario.

Figure 8 and Figure 9 analyze the constraint cost violation. These plots track the cumulative constraint violations across training episodes for all algorithms under stationary and mobile network conditions. Constraint costs arise from the exceeding predefined energy, duty cycle, fairness, and uplink utilization limits in the CMDP. SADQN and Safe-DQN maintain the lowest violation levels, confirming their ability to satisfy multi-constraint safety requirements during adaptive beamforming. Mobile scenarios exhibit slightly higher violations due to mobility-induced channel fluctuations and topology reconfiguration overhead, emphasizing the importance of safe policy regularization in dynamic underground environments.

#### 6.4.7. Stability Learning Analysis

**Figure 10:**
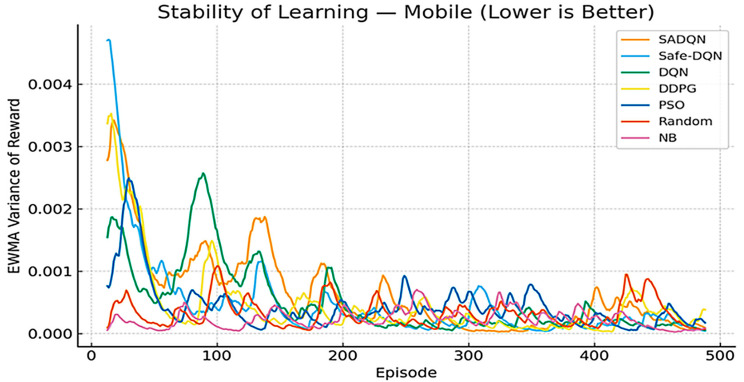
Stability learning in stationary scenario.

**Figure 11:**
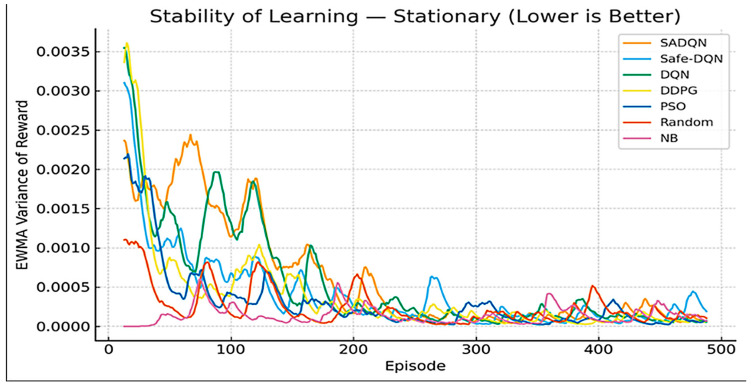
Stability learning in mobile scenario.

Figure 10 and Figure 11 present the exponential moving variance of the learning reward, serving as an indicator of training stability. SADQN and Safe-DQN maintain the lowest variance throughout training, demonstrating robust policy updates and stable reward prediction. Low-variance performance ensures a reliable convergence under fluctuating SINR and harvested-power feedback typical of underground mine channels. Higher fluctuations in DQN and PSO signify sensitivity to exploration noise and lack of embedded constraint awareness in their reward models.

## 7. Conclusions

This paper presented a Safe Adaptive Deep Q-Network (SADQN) framework for energy beamforming in LoRa-enabled RF wireless power transfer systems designed for disaster-tolerant underground mining networks. The proposed approach addresses three critical limitations of existing beamforming techniques: the absence of explicit safety mechanisms, lack of residual energy-aware node prioritization, and inadequate support for long-range, low-power communication in post-disaster underground environments. The SADQN framework formulates the beamforming problem as a constrained Markov decision process (CMDP) and employs Lagrangian relaxation with dual-variable updates to transform the NP-hard constrained optimization into a tractable problem.

Comprehensive simulation results validated the effectiveness of the proposed approach. SADQN achieved significant improvements in cumulative harvested energy: approximately 11% versus DQN, 15% versus Safe-DQN, and 40% versus PSO in mobile scenarios. The framework maintained fairness indices above 0.90, converged 27% faster than Safe-DQN and 43% faster than DQN, and exhibited 33% lower performance variance than Safe-DQN. Safe-DQN reduced constraint violations by 66% versus SADQN and 59% versus DQN, demonstrating the critical trade-off between energy maximization and safety compliance.

The framework incorporates five key adaptive mechanisms: energy-aware scoring, fairness bonus incentives, adaptive exploration, composite scoring, and fairness-aware top-K action selection. These mechanisms enable safe, efficient beamforming policies without requiring perfect channel state information. The dual-band architecture (860 MHz for WPT and 915 MHz for LoRa uplink) successfully addressed the 125+ dB power asymmetry between energy-harvesting requirements and LoRa communication sensitivity, enabling real-time voltage feedback without extensive CSI acquisition. The sequential charging strategy, which prioritizes the weakest nodes, proved particularly effective for disaster scenarios where maintaining tracking and localization capabilities is paramount.

## Figures and Tables

**Table 1:** Comparison of related work and proposed SADQN framework.

Feature	Prior EB Work [16,17,20]	Prior RL Work [21,22,25]	Energy-Aware RL [26,28]	LoRa Systems [18,19,29]	Proposed SADQN
EB	✓	X	Partial	X	✓
CSI-Free Operation	Partial	X	X	N/A	✓
Safety Constraints	X	X	X	N/A	✓
Residual Energy Awareness	X	X	Partial	X	✓
Node Prioritization (Triage)	X	X	X	X	✓
Fairness Guarantees	X	X	✓	N/A	✓
Mobility Support	Partial	✓	Partial	X	✓
Underground Propagation	X	X	X	✓	✓
Long-Range Low-Power (LoRa)	X	X	X	✓	✓
Dual-Band Architecture	X	X	X	X	✓

**Table 2:** Symbol and meaning.

Symbol	Meaning
$N_{t}$, d,λ	Number of TX antenna elements, spacing, wavelength
a(θ)	ULA steering vector at angle θ
$w_{t}$	Transmit weights (conjugate beam)
$\phi_{i,t}$	Angular drift for node-*i* at time *t*
$G_{i,t}$	Beamforming array gain towards node-*i*
$v_{i,t}\in \left[0,V_{max}\right]$	Harvested voltage at node-*i*
$E_{i,t}$, $E_{max}$	Stored energy at node-*i*, maximum storable energy
$r_{t}$	Instantaneous reward at time *t*
$J_{t}\in \left[0,1\right]$	Jain fairness over {$E_{i,t}$}
$s_{t}$	State space at time *t*
$a_{t}\in A\cup \left\{\emptyset \right\}$	Beam steering angle or idle action ∅
$c_{sw},c_{fair},c_{min},c_{duty},c_{UL}$	Constraints cost functions
λ≥0	Dual variables (Lagrange multipliers)
$\bar{r_{t}}$	Shaped reward with dual penalties
$k_{t}$	Top-*k* candidate actions
$\varepsilon_{t}$	Exploration decay
C	Complex number field
${\left(.\right)}^{H}$	Hermitian (conjugate) transpose
E	Expectation operator
γ	Discount factor in [0, 1]
Δt	Time-slot duration (s)
$\eta_{RF-DC}$	Linear RF-to-DC model efficiency
$\rho_{leak}$	RF energy storage leakage time constant (s)
$P_{load}$	Average load power during slot (W)
$\eta_{\lambda }$	Dual ascent step size for $\lambda_{j}$ updates

**Table 3:** Summary of the constraints’ notation and meaning.

Constraint	Symbol	Unit	Definition
Switching smoothness	$c_{sw,t}$	Dimensionless	Penalizes abrupt beam changes that exceed a per-step angular jump limit.
Fairness	$c_{fair,t}$	Dimensionless	Penalizes energy imbalance across nodes.
Minimum energy requirement	$c_{min,t}$	Dimensionless	Penalizes nodes below energy floor.
TX duty	$c_{duty,t}$	Dimensionless	Penalizes downlink WPT over-use vs. regulatory duty budget.
UL load (agent over-flooding)	$c_{UL,t}$	Dimensionless	Penalizes uplink feedback load exceeding per-node budget.

## Data Availability

The energy-harvesting data was enclosed in the main manuscript.
